# Beta-amyloid deposition in patients with major depressive disorder with differing levels of treatment resistance: a pilot study

**DOI:** 10.1186/s13550-017-0273-4

**Published:** 2017-03-21

**Authors:** Peng Li, Ing-Tsung Hsiao, Chia-Yih Liu, Chia-Hsiang Chen, She-Yao Huang, Tzu-Chen Yen, Kuan-Yi Wu, Kun-Ju Lin

**Affiliations:** 1 Department of Psychiatry, Chang Gung Memorial Hospital and Chang Gung University, 5. Fu-Hsing Street. Kuei Shan Hsiang, Tao-Yuan, Taiwan; 2 Department of Nuclear Medicine and Molecular Imaging Center, Chang Gung Memorial Hospital, Tao-Yuan, Taiwan; 3 Department of Medical Imaging and Radiological Sciences and Healthy Aging Research Center, Chang Gung University, Tao-Yuan, Taiwan; 4 Department of Nuclear Medicine and Center for Advanced Molecular Imaging and Translation, Chang Gung Memorial Hospital, No. 5, Fuxing St., Guishan Dist, Taoyuan City, 333 Taiwan

**Keywords:** Major depressive disorder, Treatment resistance, Amyloid, ^18^F-Florbetapir (AV-45/Amyvid), Alzheimer’s disease, Dementia

## Abstract

**Background:**

Lack of treatment response in patients with late-life depression is common. The role of brain beta-amyloid (Aβ) deposition in treatment outcome in subjects with late-life depression remains unclear. The present study aimed to investigate brain Aβ deposition in patients with major depressive disorder (MDD) with differing treatment outcomes in vivo using ^18^F-florbetapir imaging.

This study included 62 MDD patients and 18 healthy control subjects (HCs).We first employed the Maudsley staging method (MSM) to categorize MDD patients into two groups according to treatment response: mild treatment resistance (*n* = 29) and moderate-to-severe treatment resistance (*n* = 33).The standard uptake value ratio (SUVR) of each volume of interest was analysed, and voxel-wise comparisons were made between the MDD patients and HCs. Vascular risk factors, serum homocysteine level, and apolipoprotein E (ApoE) genotype were also determined.

**Results:**

The MDD patients with moderate-to-severe treatment resistance had higher ^18^F-florbetapir SUVRs than the HCs in the parietal region (*P* < 0.01). Voxel-wise comparisons further demonstrated elevated SUVRs in MDD patients with moderate-to-severe treatment resistance in the precuneus, parietal, temporal, and occipital regions. The MDD patients with mild treatment resistance were found to have increased ^18^F-florbetapir uptake mainly in the left frontal and parietal regions as compared with the HCs. In addition, voxel-to-voxel correlation analysis showed that brain Aβ deposition was correlated positively with MSM score in the occipital region. ^18^F-florbetapir SUVRs were correlated negatively with Mini Mental Status Examination (MMSE) score in the sample of all MDD patients (*r* = −0.355, *P* = 0.005).

**Conclusions:**

This study provided preliminary evidence that region-specific Aβ deposition was present in some (but not all) MDD patients, especially in those with moderate-to-severe treatment resistance, and their depressive symptoms may represent prodromal manifestations of Alzheimer’s disease (AD). Depressive symptomatology in old age, particularly in subjects with a poor treatment response, may underscore early changes of AD-related pathophysiology.

## Background

Late-life depression is common in the elderly and usually accompanies cognitive and functional decline, which may result in increased mortality and disability. More than half of patients with late-life depression were found to respond only partially to initial first-line pharmacologic treatment [1, 2]. Impaired cognitive function is known to be frequently associated with response to treatment for depression [2]. Mounting evidence from many epidemiologic studies has indicated that a lifetime history of major depression is associated with an increased risk of developing dementia, including Alzheimer’s disease (AD) [3–5]. One postmortem study [6] showed that AD patients with a lifetime history of major depression had more pronounced amyloid plaque and neurofibrillary tangles as compared with AD patients without a history of depression. Non-invasive positron emission tomography (PET) imaging to assess brain beta-amyloid (Aβ) deposition, one of the hallmarks of AD pathology, permits direct assessment of brain AD pathology in vivo. Some studies have shown that a lifetime history of major depression is associated with brain Aβ deposition [7, 8].

Notably, some recent studies have provided evidence to show that depressive symptoms in old age might be affected by brain Aβ pathology. One large-sample prospective study [9] focused on cognitively normal older adults and found that an elevated Aβ burden increased the risk of developing clinically significant depressive symptoms during follow-up in the preclinical stage of AD. In addition, a recent review article [10] proposed that brain Aβ accumulation may be an etiologic factor affecting the emergence of late-life depression and the level of treatment resistance by interfering with the brain mood-related frontolimbic network. However, at present, the association between brain Aβ deposition and treatment outcome in late-life depression is not well understood.

Treatment resistance in patients with depression is a significant clinical phenomenon and has both personal and social impacts owing to cognitive impairment, poor functioning and increased mortality [11]. Currently, treatment resistance in patients with depression is defined as failure to achieve remission following two trials of antidepressant treatment, but there is no consistent operational definition [12]. The Maudsley staging method (MSM) [13, 14] was recently developed in order to incorporate additional factors related to depressive disorder itself, in addition to a number of failed treatment trials. The MSM results in a score between 3 and 15 and allows classification of treatment resistance into three categories (mild, moderate and severe).

Therefore, we aimed in the present study to investigate brain Aβ deposition in MDD patients without dementia with differing levels of treatment resistance. We hypothesized that greater resistance to treatment for depression would be related to greater amyloid deposition in MDD patients. In the current study, we used ^18^F-florbetapir PET to investigate (1) brain Aβ deposition in MDD patients with differing levels of treatment resistance and (2) the relationship between Aβ burden and treatment resistance, in order to determine whether treatment resistance is associated with amyloid deposition in MDD patients.

## Methods

### Subjects and protocol

This study included 62 MDD patients without dementia and 18 healthy controls (HCs). A consecutive series of MDD patients was recruited from the geriatric psychiatric outpatient clinic at Chang Gung Memorial Hospital (CGMH). To be eligible for inclusion, patients had to be aged over 50, had to be diagnosed with MDD according to the DSM-IV criteria, had to have a clinical dementia rating (CDR) of 0 or 0.5, and must have been functioning well in activities of daily living. The control subjects were all confirmed to have a lifetime absence of psychiatric illness. The exclusion criteria for all subjects included clinically significant medical diseases or neurological diseases, alcohol or other substance dependence within the past year, and a current severe risk of suicide or psychotic depression. None of the participants met the National Institute of Neurological and Communicative Disorders and Stroke and the Alzheimer’s Disease and Related Disorders Association (NINCDS-ADRDA) criteria for probable AD or the DSM-IV criteria for dementia. In addition, three Mini Mental Status Examination (MMSE) values representing different educational levels were used to exclude subjects in this study [15, 16], i.e. less than 16 indicated illiteracy, less than 21 indicated grade school literacy, and less than 24 indicated junior high school and higher education literacy. These cutoff values have a validated sensitivity of 100% for dementia [16]. All subjects were evaluated by the same board-certified geriatric psychiatrist to examine their clinical characteristics.

The MDD patients were evaluated in terms of lifetime presence and course of major depressive episodes according to the DSM-IV criteria, treatment history, and severity of depression. Diagnosis and treatment of subjects with a lifetime history of MDD were also assessed using available medical information, including charts and information obtained from the treating physician. The clinical characteristics of MDD, including age of onset, number of major depressive episodes, treatment and response history, and presence of late-onset MDD (cutoff age 60 years) were recorded for further analysis. All eligible subjects were subjected to ^18^F-florbetapir PET imaging. We also measured the serum homocysteine level and assessed vascular risk factors as defined by the Framingham stroke risk score (FSRS). The ApoE genotype of all subjects was determined by PCR (polymerase chain reaction) amplification of genomic DNA. The MMSE score was taken to represent global cognitive function, and the CDR Sum of Boxes (CDR-SB) was used to characterize cognitive and functional performance. The protocol was approved by the institutional review board of CGMH. Written informed consent was obtained from all subjects prior to enrollment in the study.

### Measures and categories of treatment resistance

As treatment resistance in depression involves many dimensions, we assessed the degree of treatment resistance using a points-based staging model, the MSM [13]. The MSM incorporates three main factors: treatment (i.e. number of antidepressant treatment failures and whether augmentation or electroconvulsive therapy had been used), severity of symptoms, and duration of presenting episode. The MSM score was used as a covariate in subsequent analyses. Staging of treatment resistance was also performed according to three categories of severity: mild (score = 3–6), moderate (score = 7–10), and severe (score = 11–15). In this study, only five MDD patients were classified into the group of subjects with severe treatment resistance based on MSM score. Due to the limited number of patients with severe treatment resistance in our sample, we categorized the MDD patients into two groups overall: subjects with mild treatment resistance (MSM score ≤6) and subjects with moderate-to-severe treatment resistance (MSM score ≥7). This method categorized most patients with only one to two failures of antidepressant treatment into the mild treatment resistance group.

### Amyloid PET acquisition

Radiosynthesis and acquisition of ^18^F-florbetapir PET imaging have been described as before [17, 18]. In summary, a ^18^F-florbetapir PET scan was performed using a Biograph mCT PET/CT system (Siemens Medical Solutions, Malvern, PA). A 10-min PET scan was acquired at 50 min post-injection of 380 ± 18 MBq of ^18^F-florbetapir. The 3-D OSEM reconstruction algorithm (four iterations, 24 subsets; Gaussian filter 2 mm, zoom 3) was applied with CT-based attenuation correction, and scatter and random corrections, and that led to reconstructed images with a matrix size of 400 × 400 × 148 and a voxel size of 0.68 × 0.68 × 1.5 mm.

### Image analysis

The image analysis software of PMOD (version 3.3; PMOD Technologies Ltd, Zurich, Switzerland) was used for all image process and analysis. Each PET image was spatially normalized to the Montreal Neurological Institute (MNI) space using a MR-based spatial normalization. Eight volumes of interest (VOIs), including the whole cerebellum, frontal, anterior cingulate, posterior cingulate, precuneus, parietal, occipital, and temporal areas, were selected based on the modified automated anatomic labelling (AAL) atlas [19]. The voxel-wise standardized uptake value ratio (SUVR) images were calculated using the whole cerebellum reference region, and regional SUVR was measured from the mean SUVR of each VOI. The global cortical SUVR was calculated from the average SUVR of seven cerebral cortical VOIs for further analysis.

### Voxel-wise analysis

The SPM12 software package (Wellcome Department of Cognitive Neurology, Institute of Neurology, London, UK) was applied for voxel-wise imaging analysis implemented in Matlab 2010a (MathWorks Inc., Natick, MA). Smoothing using an isotropic Gaussian kernel of 8 mm FWHM (full-width at half-maximum) was performed on the previously spatially normalized SUVR images of ^18^F-florbetapir. To compare the HCs and the two MDD subgroups, two-sample *t* tests were conducted on the amyloid SUVR images, and SPM t-maps were examined with an uncorrected threshold of *P* < 0.01 and an extent threshold of 100 voxels.

### Statistical analysis

Data are expressed as means ± SD or as absolute numbers with proportions for descriptive statistics. The regional SUVRs of the ^18^F-florbetapir PET images were compared region by region individually using the non-parametric Kruskal-Wallis test with Dunn’s multiple comparison post hoc analysis for group comparisons between the HCs, the MDD group with mild treatment resistance, and the MDD group with moderate-to-severe treatment resistance. Pearson’s correlation was used to evaluate the correlations between the global ^18^F-florbetapir SUVR and the MMSE score in the MDD patients. Multiple linear regression analysis was used to further evaluate the association of ^18^F-florbetapir binding with cognitive function in the MDD group after controlling for age, sex, educational level, ApoE ε4 genotype, and FSRS. A *P* value of 0.05 was taken as the threshold for statistical significance in each test.

## Results

### Clinical characteristics of each group

This study included 62 MDD patients and 18 HCs. Among the MDD patients, 29 (46.8%) were categorized into the mild treatment resistance group and 33 (53.2%) had moderate-to-severe treatment resistance. Table 1 shows the demographic and clinical characteristics of the HCs and the two groups of MDD patients with mild and moderate-to-severe treatment resistance, respectively. These groups did not differ significantly in terms of age, gender, years of education, ApoE ε4 genotype, homocysteine level, or FSRS. All MDD patients had significantly lower MMSE scores than the HCs. The MDD patients with moderate-to-severe treatment resistance had more depressive episodes and higher CDR-SB scores as compared with the MDD patients with mild treatment resistance.

**Table 1:** Demographic and clinical characteristics of the healthy controls (HCs) and patients with major depressive disorder (MDD) with differing levels of treatment resistance

Characteristic	HCs	MDD patients		*P* value
		Mild treatment resistance	Moderate-to-severe treatment resistance	
No. of subjects	18	29	33	
Age (years)				0.537
Mean ± SD	68.6 ± 5.5	66.6 ± 6.8	65.0 ± 5.7	
Median (IQR)	68 (64.8–73.0)	65.0 (61.5–71.0)	66.0 (61.5–68.5)	
Female gender, *n* (%)	11 (61.0)	22 (75.9)	25 (75.8)	0.47
Education (years)				0.065
Mean ± SD	9.8 ± 3.9	7.2 ± 4.2	8.7 ± 4.0	
Median (IQR)	12 (6.0–12.5)	6.0 (6.0–10.5)	6.0 (6.0–12.0)	
HAM-D				<0.001***
Mean ± SD	2.0 ± 1.5	4.9 ± 3.5^a^*	10.4 ± 6.5^a^***^,b^**	
Median (IQR)	1.5(1.0–2.3)	3.0(2.0–7.5)	8.0(6.0–14.5)	
MMSE				<0.002**
Mean ± SD	27.3 ± 1.8	25.2 ± 2.4^a^**	24.7 ± 3.1^a^**	
Median (IQR)	28 (26.8–28.3)	26.0 (24.0–27.0)	25.5 (22.5–27.0)	
ApoE ε4, *n* (%)	2 (11.1)	5 (17.2)	9 (27.3)	0.347
FSRS				0.998
Mean ± SD	8.5 ± 1.9	8.7 ± 4.4	8.6 ± 4.1	
Median (IQR)	9.0 (7.0–10.0)	10.0 (4.5–12.5)	9.0 (5.5–12)	
Homocysteine (μmol/l)				0.299
Mean ± SD	8.6 ± 1.8	8.9 ± 2.6	9.8 ± 2.9	
Median (IQR)	8.7 (7.3–9.5)	8.6 (7.0–10.7)	9.4 (7.6–11.1)	
Age at onset (years)	–			0.122
Mean ± SD	–	57.2 ± 12.5	53.8 ± 9.9	
Median (IQR)	–	57.0 (49.5–65.5)	53.0 (49.0–60.0)	
Duration of MDD (years)	–			0.183
Mean ± SD	–	9.3 ± 9.5	11.3 ± 9.0	
Median (IQR)	–	8.0 (1.5–11.5)	10.5 (5.0–13.0)	
Number of depressive episodes	–			0.005**
Mean ± SD	–	1.6 ± 0.6	2.5 ± 1.4^b^**	
Median (IQR)	–	1.0 (1.0–2.0)	2.0 (2.0–3.0)	
Late-onset MDD, *n* (%)	–	14 (48.3)	9 (33)	0.088
MSM score	–			<0.001***
Mean ± SD	–	4.1 ± 1.0	8.4 ± 2.4^b^***	
Median (IQR)	–	4.0 (3.0–5.0)	9.0 (7.0–10.0)	
CDR 0.5, *n* (%)	–	12 (41.4)	25 (80.6)^b^**	0.002**
CDR-SB				0.001**
Mean ± SD		0.3 ± 0.5	1.0 ± 0.7b**	
Median (IQR)	–	0 (0.0–0.5)	1 (0.3–1.5)	

*HAM-D* 17-item Hamilton depression rating scale, *FSRS* Framingham stroke risk score, *MMSE* Mini Mental Status Examination, *ApoE ε4* apolipoprotein E ε4 carrier, *MSM* Maudsley staging method, *CDR* Clinical Dementia Rating scale, *CDR-SB* Clinical Dementia Rating–Sum of Boxes

^a^Significant difference as compared with HCs: *P < 0.05, **P < 0.01, ***P < 0.001

^b^Significant difference as compared with MDD patients with mild treatment resistance: *P < 0.05, **P < 0.01, ***P < 0.001

### Treatment resistance and Aβ deposition

Table 2 shows the ^18^F-florbetapir SUVRs in seven cortical VOIs and the global cortex in the HCs and the MDD patients with mild and with moderate-to-severe treatment resistance. There were significant differences in the ^18^F-florbetapir SUVR in the parietal region between the three groups (*P* = 0.029). Post hoc analysis showed significant differences between the MDD patients with moderate-to-severe resistance and the HCs (*P* < 0.01). Although not significant, the global cortical SUVR in the three groups seemed to be ordered as follows: moderate-to-severe resistance > mild resistance > HCs.

**Table 2:** ^18^F-florbetapir SUVRs in the healthy controls (HCs) and patients with major depressive disorder (MDD) with differing levels of treatment resistance in seven cortical VOIs and the global cortex

Region	HCs	MDD patients		*P* value
		Mild treatment resistance	Moderate-to-severe treatment resistance	
Frontal				0.365
Mean ± SD	1.09 ± 0.09	1.12 ± 0.11	1.11 ± 0.15	
Median (IQR)	1.07 (1.04–1.16)	1.09 (1.06–1.15)	1.07 (1.03–1.15)	
Anterior cingulate				0.606
Mean ± SD	1.21 ± 0.11	1.24 ± 0.12	1.21 ± 0.15	
Median (IQR)	1.18 (1.13–1.30)	1.24 (1.16–1.33)	1.22 (1.11–1.29)	
Posterior cingulate				0.251
Mean ± SD	1.31 ± 0.12	1.31 ± 0.16	1.36 ± 0.16	
Median (IQR)	1.28 (1.21–1.43)	1.29 (1.24–1.41)	1.35 (1.27–1.44)	
Occipital				0.284
Mean ± SD	1.15 ± 0.08	1.18 ± 0.08	1.21 ± 0.13	
Median (IQR)	1.16 (1.09–1.21)	1.17 (1.14–1.23)	1.19 (1.12–1.24)	
Parietal				0.032*
Mean ± SD	1.01 ± 0.08	1.08 ± 0.11	1.11 ± 0.15^a^*	
Median (IQR)	1.03 (0.94–1.06)	1.06 (1.00–1.17)	1.08 (1.02–1.14)	
Precuneous				0.201
Mean ± SD	1.02 ± 0.08	1.06 ± 0.1	1.10 ± 0.17	
Median (IQR)	1.02 (0.95–1.09)	1.04 (1.00–1.10)	1.06 (0.99–1.13)	
Temporal				0.693
Mean ± SD	1.03 ± 0.06	1.02 ± 0.07	1.03 ± 0.12	
Median (IQR)	1.02 (1.00–1.07)	1.01 (0.99–1.04)	1.00 (0.95–1.08)	
Global				0.52
Mean ± SD	1.13 ± 0.07	1.15 ± 0.09	1.16 ± 0.13	
Median (IQR)	1.11 (1.07–1.16)	1.13 (1.11–1.19)	1.14 (1.09–1.18)	

^a^Significant difference as compared with HCs: *P < 0.05

**Fig. 1:**
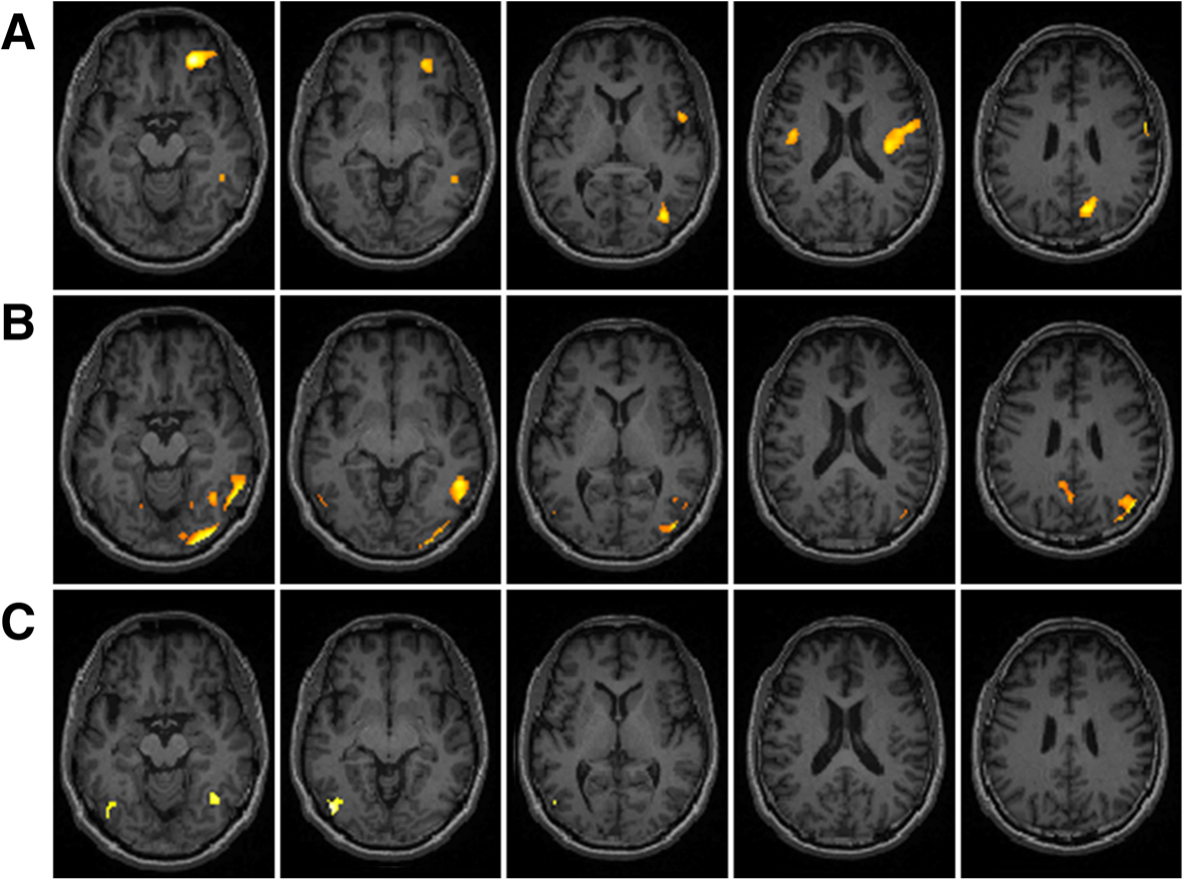
Spatial distribution of increased ^18^F-florbetapir SUVRs in the MDD patients with differing levels of treatment resistance as compared with the healthy controls (HCs), as examined by statistical parametric mapping (SPM) analysis, with an uncorrected *P* < 0.01 and clusters consisting of a minimum of 100 contiguous voxels, which were considered to indicate a significant difference. SPM results showing relatively high amyloid loading in MDD patients with mild treatment resistance versus controls (**a**); MDD patients with moderate-to-severe treatment resistance versus controls (**b**); and MDD patients with moderate-to-severe treatment resistance versus MDD patients with mild treatment resistance (**c**) (*P* < 0.01, uncorrected, extend voxel *k* = 100)

The SPM analyses are presented in Fig. 1. The results showed that the MDD patients with moderate-to-severe resistance had significantly higher ^18^F-florbetapir SUVRs than the HCs in the precuneous, parietal, temporal, and occipital areas. The MDD patients with mild resistance were observed to have significantly higher ^18^F-florbetapir binding than the HCs in the frontal, parietal, and occipital areas. As compared with the patients with mild resistance, those with moderate-to-severe resistance were observed to have higher ^18^F-florbetapir SUVRs in the temporal and occipital cortex areas.

**Fig. 2:**
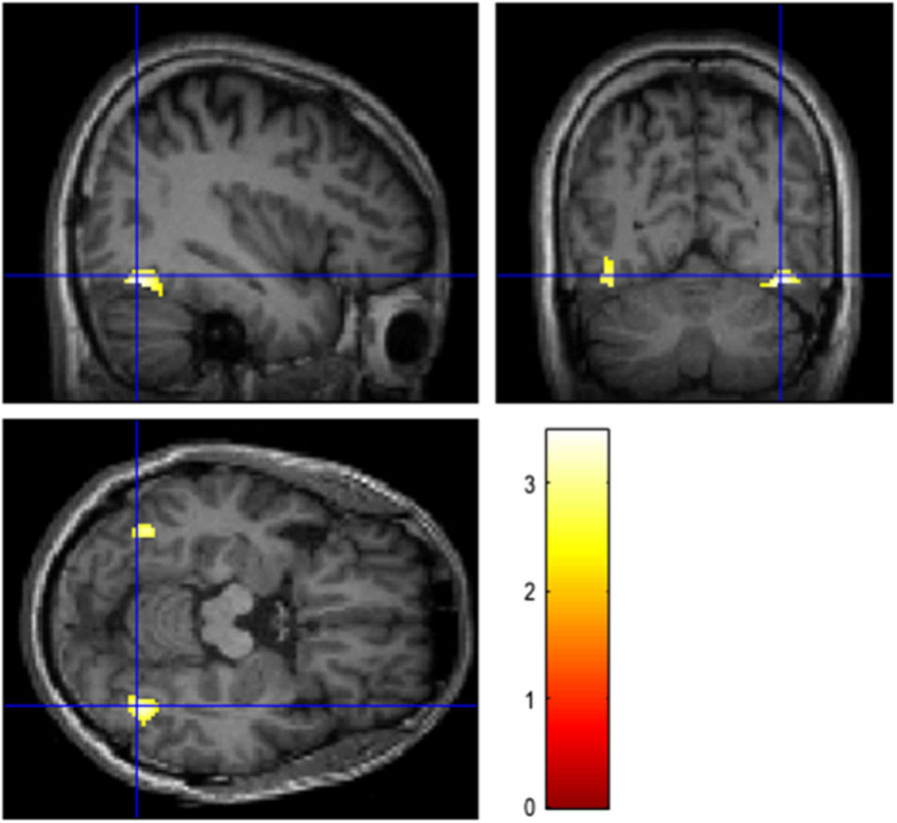
Voxel-by-voxel correlation between brain amyloid loading and Maudsley staging method score

To assess the relationship between Aβ burden and the level of treatment resistance, we examined voxel-by-voxel the correlation between Aβ load and MSM score. MSM score was found to be positively significantly correlated with Aβ burden over the occipital region (Fig. 2).

### Aβ deposition and cognitive function in MDD patients

**Fig. 3:**
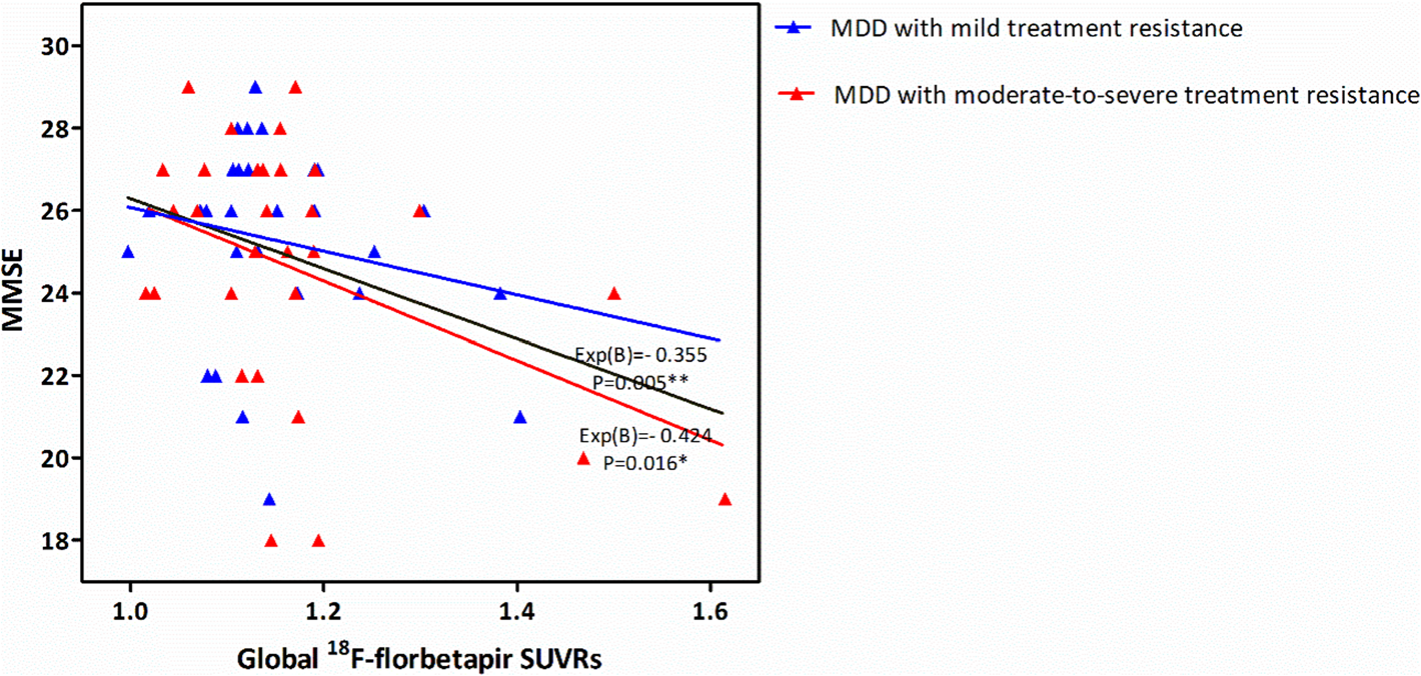
Relationship between global ^18^F-florbetapir SUVR and MMSE score in the MDD patients

The global cortical SUVR was found to be significantly negatively correlated with the MMSE score (*r* = −0.355, *P* = 0.005) in the sample of all MDD patients. The global cortical SUVR was also significantly negatively correlated with MMSE score (*r* = −0.424, *P* = 0.016) in the MDD group with moderate-to-severe treatment resistance (Fig. 3). The negative correlation remained significant in multiple regression analyses after controlling for age, gender, educational level, homocysteine level, and FSRS among the whole MDD group (*β* = −8.311, *t* = −3.024, *P* = 0.004) and the MDD subjects with moderate-to-severe treatment resistance (*β* = −9.425, *t* = −2.725, *P* = 0.012).

## Discussion

Although a growing number of clinical studies has indicated an association between a history of depression and brain Aβ accumulation, there have been few studies focusing on Aβ deposition in MDD patients with differing treatment outcomes. To our knowledge, this was the first study to investigate brain Aβ load in middle-aged to elderly MDD patients with different treatment outcomes in vivo using ^18^F-florbetapir imaging. In this study, we first employed the MSM score to categorize MDD patients into two groups: mild treatment resistance and moderate-to-severe treatment resistance. Under the circumstance of no differences in demographic characteristics between groups, the MDD patients with moderate-to-severe resistance exhibited higher ^18^F-florbetapir binding than the HCs in the parietal region according to VOI analysis. Further analysis of the parametric ^18^F-florbetapir images was conducted to examine differences in regional SUVRs between groups. The MDD patients with moderate-to-severe treatment resistance had increased ^18^F-florbetapir uptakes in the precuneus, parietal, temporal, and occipital regions; also, the patients with mild treatment resistance were found to have increased ^18^F-florbetapir uptakes in mainly the left frontal and parietal regions as compared with the HCs. In addition, voxel-to-voxel correlation analysis showed that brain Aβ deposition in the occipital region was positively correlated with the MSM score. ^18^F-florbetapir SUVRs were negatively correlated with the MMSE score in the sample of all MDD patients.

Impaired cognitive function in late-life depression and MRI white-matter hyperintensities have been frequently shown to be associated with the outcome of clinical treatment for depression [2, 15]. Our previous study [19] showed that MDD patients with mild cognitive impairment (MCI) had heterogeneously elevated ^18^F-florbetapir retention. The MDD patients with amnestic MCI had similar regional distributions of Aβ burden to the early AD patients, further suggesting a risk of developing AD in the future. In the present study, the MDD patients with moderate-to-severe treatment resistance were also found to have an Aβ spatial distribution similar to those of patients with MCI or early AD [20–24]. In addition, cognitive function as assessed by the MMSE score was negatively correlated to amyloid deposition in this group of MDD patients. Collectively, the characteristics described above suggested that the MDD patients with moderate-to-severe treatment resistance might be at the preclinical or even prodromal stage of AD. A recent study using ^11^C-Pittsburgh Compound B (PIB) imaging [25] found that cross-sectional depressive symptoms were positively correlated with the mean cortical Aβ deposition in cognitively normal subjects with a higher cerebral Aβ burden, but not in subjects with low and medium Aβ burdens. The main increase in Aβ pathology in subjects with a high cerebral Aβ burden was localized to the precuneus/posterior cingulate cortex as compared with subjects with a medium Aβ burden. Collectively, recent findings have implied that more aggressive depressive symptoms in later life might be related to brain region-specific Aβ deposition and may indicate that these patients are at risk of preclinical AD.

Previous studies have suggested that structural and functional abnormalities in the brain network may contribute to resistance to antidepressant treatment in depressed older patients without dementia [26, 27]. In one study which investigated depression in AD, depressed AD patients showed decreased functional connectivity between the anterior cingulate cortex, the right lingual gyrus, and the right occipital lobe compared to non-depressed AD patients [28]. In another study, during treatment of depression, remitters to escitalopram showed a significant tendency to modify resting-state activity in the occipital cortex. Conversely, non-remitters showed much lower levels of significant changes. It suggested that treatment response might be associated with activity in the occipital resting-state network [29]. In the present study, compared with the MDD patients with mild treatment resistance, the patients with moderate-to-severe resistance had greater ^18^F-florbetapir binding in the temporal and occipital regions. Moreover, MSM score was found to be significantly correlated with Aβ burden over the occipital region. Therefore, our findings also suggested that treatment response of depression might be associated with local or distant damage from Aβ pathology occurred in these brain regions.

Notably, the MDD patients with mild treatment resistance exhibited elevated Aβ loads, mainly in the left frontal and parietal areas. A recently published study from the Alzheimer’s Disease Neuroimaging Initiative (ADNI) [30] focused on a population of Aβ-positive MCI subjects and found that subsyndromally depressed subjects had elevated amyloid loads in the left medial frontal, left superior temporal, and left parietal regions as compared with non-depressed subjects. In another recent study [8], amnestic MCI patients with a lifetime MDD history were compared with amnestic MCI patients without a lifetime MDD history. The regions with higher Aβ depositions in the patients with a lifetime MDD history included the bilateral prefrontal cortex and some regions in the right temporal area. These brain regions affected by Aβ pathology comprise and connect with the prefrontal network known to be related to depressive disorder [31]. In our study, voxel-wise analyses showed that the MDD patients with moderate-to-severe resistance had significantly higher ^18^F-florbetapir SUVRs than the HCs in the precuneous, parietal, temporal, and occipital regions, but not in the frontal area. Thus, we further performed subgroup analyses for amyloid positive group of the MDD patients. The cerebral amyloid positive cutoff point (global SUVR 1.178) was determined using independent data obtained from clinically diagnosed AD patients in a previous study by our research team [24]. The subgroup analyses found that the MDD patients with moderate-to-severe resistance (*n* = 8) also had significantly higher ^18^F-florbetapir SUVRs than the HCs in the frontal area and showed the similar regional distribution of increased ^18^F-florbetapir uptakes (*n* = 9) (data not shown). The preliminary findings of our study also suggested that depressive symptomatology might be related to Aβ deposition in the frontal area. Some researchers have hypothesized that Aβ accumulation might lead to pathophysiologic events that impair the brain frontolimbic or frontostriatal circuitry and predispose the patient to treatment-resistant depressive symptoms before the emergence of clinically significant cognitive impairment [15, 16, 25, 32]. Based on the collective evidence mentioned above, we speculated that the brain regions of the mood-related prefrontal network affected by Aβ pathology might be linked to the clinical presentation of late-life depression.

Greater amyloid burden had been demonstrated to be correlated to lower cognitive performance in cognitive normal older individuals [33, 34]. The present study was consistent with the previous results and found a negative correlation between ^18^F-florbetapir SUVRs and the MMSE score in the sample of all MDD patients. We further conducted multiple regression analyses, and the negative correlation remained significant among the whole MDD group and the MDD patients with moderate-to-severe treatment resistance. Impaired cognitive function in late-life depression has been frequently related to the outcome of clinical treatment for depression [2]. It suggested the potential association between brain Aβ deposition and treatment outcome in late-life depression. Given small sample size in this study, there was no significant difference of global SUVRs between two MDD groups. However, the negative correlation between ^18^F-florbetapir SUVRs and the MMSE score was noted when the MDD subjects with moderate-to-severe treatment resistance were included. It implied the relationship between Aβ loads and cognition in MDD patients was driven by the subjects with moderate-to-severe treatment resistance who had relatively higher Aβ accumulation and lower MMSE score, compared to the patients with mild treatment resistance. Most importantly, region-specific brain Aβ depositions similar to early AD patterns were observed in the MDD patients with moderate-to-severe treatment resistance. Therefore, this group of MDD subjects may be at greater risk of developing AD in the future.

More and more evidence is being produced that supports the hypothesis that depressive symptomatology in old age, in persons both with and without MCI, may be an early symptom of an underlying AD neuropathological mechanism [25, 32, 35–39]. Furthermore, one study [40] showed that patients with both MCI and depression are at greater risk of developing AD than those with MCI alone. In our previous study, patients without dementia with lifetime MDD had regionally higher ^18^F-florbetapir SUVRs in the parietal and precuneus cortex areas [7]. Meanwhile, the results of the present study showed that the MDD patients with a higher level of treatment resistance had regionally higher ^18^F-florbetapir SUVRs in the parietal cortex area. Previous evidence demonstrated that a higher Aβ burden in this area is linked to AD conversion and was found among patients with early AD [41–44]. Therefore, this group of MDD subjects may be at greater risk of developing AD, and their cognitive function should be followed up in a clinical setting. The results of this study suggested that late-life depression in some (but not all) patients might be related to disruption of mood-related frontolimbic networks by Aβ deposition, which may cause a vicious circle, further worsening the outcome of depression and impairing cognitive function. However, results should be interpreted with caution due to small subject number of this preliminary study limitation. The inspection was done using a less strict statistical cutoff point (*P* < 0.01, uncorrected). Future work with more subjects could overcome the methodological issue encountered during our study. Although the present study suggests that amyloid accumulation may be associated with early signs of cognitive decline, longitudinal studies are required to understand how likely and how long it will be before such subjects progress to more serious levels of impairment. Future studies are needed to examine whether Aβ deposition is a factor that directly moderates treatment response in late-life MDD patients.

### Limitations

The present study had several limitations. First, the sample size used in the study was relatively small; thus, our findings may not be relevant to other populations or groups. Given the relatively small sample size, we were unable to classify the subjects into amyloid positive/negative or with/without MCI groups. Second, we included cases only from an outpatient setting to form the MDD group, which could explain the relatively small number of patients with severe treatment resistance; thus, the results are not necessarily representative of the larger population of patients with MDD. The small sample size might lead to a result that may not be of sufficient power to detect differences in regional and global amyloid burdens between subjects with severe, moderate, and mild treatment resistance and the control group. Third, the MSM was developed using a sample group that included in the main MDD patients with severe treatment resistance. The authors also suggested that the MSM might carry the potential for non-generalizability of findings to less severe MDD or outpatient populations [13]. Other limitations of this method also exist: (1) the number of augmentation strategies and combinations of antidepressants are not included in the dimension of treatment failure; (2) the duration of illness is arbitrarily divided into three categories; (3) use of the chart review methodology causes potential recall bias; and (4) there is a lack of information about psychiatric/somatic comorbidity (operationalized by criteria) and previous psychotherapies. However, compared with other treatment resistance staging methods, the MSM is user friendly and enables prediction of clinical outcome after long-term follow-up [14]. Fourth, as this study employed a cross-sectional design, causality was difficult to establish. While it would be premature to draw definitive conclusions from this analysis, our findings may be useful as pilot data for future studies that include longitudinal follow-up and more representative cohorts. Finally, the MDD patients had received various antidepressant and augmentation treatments over their lifetime before they were recruited into this cross-sectional imaging study, and it was difficult to precisely estimate the lifetime cumulative dosages of antidepressants. The potential effects of antidepressant treatment on Aβ deposition and regional distribution are unknown. Future studies should be carefully designed to assess the effects of medications on amyloid binding through longitudinal follow-up.

## Conclusions

The present study highlighted differences in region-specific brain Aβ depositions in middle-aged to elderly MDD patients with differing levels of treatment resistance. Regional patterns of early AD pathology were observed in the MDD patients with moderate-to-severe treatment resistance. The patients with mild treatment resistance exhibited elevated Aβ loads, mainly in the left frontal and parietal areas. Such depressive symptoms in old age may potentially represent either prodromal manifestation of AD or an independent process interacting with underlying AD-related pathophysiology. Our findings may have clinical relevance, in that treatment response in patients with late-life depression could predict brain AD pathology and aid clinicians in identifying patients in need of vigilant follow-up to assess cognitive function.
